# Maternal antibody-mediated elimination of a Puumala hantavirus outbreak in a bank vole colony

**DOI:** 10.1371/journal.ppat.1013693

**Published:** 2026-05-29

**Authors:** Stephan Drewes, Julia Wyszkowska, Ewa Jaromin, Joanna Hajduk, Ilona Onik, Mateusz Konczal, Krystyna Lach, Barbara Bober-Sowa, Katarzyna Baliga-Klimczyk, Edyta T. Sadowska, Rainer G. Ulrich, Paweł Koteja

**Affiliations:** 1 Institute of Novel and Emerging Infectious Diseases, Friedrich-Loeffler-Institut, Federal Research Institute for Animal Health, Greifswald-Insel Riems, Germany; 2 Faculty of Biology, Institute of Environmental Sciences, Jagiellonian University, Kraków, Poland; 3 Faculty of Biology, Evolutionary Biology Group, Adam Mickiewicz University in Poznań, Poznań, Poland; 4 Partner site Hamburg-Lübeck-Borstel-Riems, German Center of Infection Research (DZIF), Greifswald-Insel Riems, Germany; NIAID: National Institute of Allergy and Infectious Diseases, UNITED STATES OF AMERICA

## Abstract

Bank voles (*Myodes glareolus* syn. *Clethrionomys glareolus*) are frequently used as an animal model in ecological and biomedical studies and are an important reservoir of viral and bacterial zoonotic pathogens, e.g., Puumala hantavirus (PUUV). Here we describe an accidental PUUV outbreak in a large bank vole laboratory colony caused by an accidental introduction of infected wild-trapped bank voles, and the successful eradication of the virus. The eradication plan was based on results of previous studies showing that maternal antibodies (MatAb) protect the young from infection for up to 40 days after weaning, four weeks longer than the estimated duration of PUUV infectivity in the environment. After ensuring that most animals were infected, 620 pairs were mated on the same day. Only females that showed PUUV-specific antibodies and produced offspring within 26 days after mating were retained. All individuals of the parental generation were euthanized before the last weaning. The weaned offspring were moved to individually ventilated cages (IVC) and repeatedly tested for the presence of PUUV-specific antibodies and RNA. A few infected or suspect animals were euthanized. The animals were then mated (in IVC) and, after producing grand-offspring generation, euthanized and tested for PUUV RNA in the lungs. No PUUV RNA was detected, and no animals showed PUUV-specific antibodies in subsequent generations. The successful clearance confirmed the protective efficiency of PUUV-specific MatAb. The procedure for PUUV clearance in the bank vole colony may represent a blueprint for similar approaches in valuable colonies of other rodents infected by similar pathogens.

## Author summary

In 2006, we started a unique long-term experiment on the bank vole, a common European rodent. Our goal was to study how animals adapt to different challenges—a process known as adaptive radiation. We established 16 vole lines: four control lines and others selected for specific behavioural and physiological traits. Over the years, this colony became a valuable model for studying evolution, physiology, and behaviour. Unfortunately, the colony became infected with Puumala hantavirus. The virus infects voles without causing obvious disease, but can cause serious zoonotic illness in humans, for which no specific medical treatment is available. At first, it seemed that the entire colony would have to be destroyed—a loss of thousands of animals and many years of research. However, we took advantage of natural protection: young voles born to infected mothers are temporarily protected by maternal antibodies. By carefully planning breeding, isolation, and testing, we created conditions in which the virus lost its infectivity before the young lost their protection. This simple yet challenging approach worked—we saved the colony. Because many animals respond to viruses in similar ways, our method may help rescue other valuable research populations without complex procedures such as embryo transfer or cross-fostering.

## Introduction

Model animals characterized by unique traits are an invaluable and extensively used research tool in both basic and applied life sciences. Their great value has typically required enormous time and financial investment. However, laboratory colonies are endangered by infectious pathogens that may be difficult or seemingly impossible to eliminate, and hence may cause a drastic reduction of the gene pool or even loss of the entire colony. Even if the pathogens are not harmful to the host animals, veterinary or sanitary authorities can order elimination of the entire colony if the pathogen poses a hazard to people [1–4]. Therefore, efficient methods of eradicating such pathogens can be invaluable. Here we describe the successful eradication of Puumala hantavirus (PUUV; *species Orthohantavirus puumalaense*) from a unique colony of bank voles (*Myodes glareolus*, syn. *Clethrionomys glareolus*) comprising 16 genetically polymorphic lines of animals from a multidirectional selection experiment [5,6].

The bank vole is a common rodent widely used as a model species in ecological research (e.g., a classical monograph [7] or recent studies [8–13]) and in biomedical studies [14–16]. In the natural environment, the bank vole is a reservoir of several zoonotic pathogens, e.g., *Leptospira* spp. and PUUV [17,18], but also most likely non-zoonotic agents, e.g., bank vole polyomavirus and bank vole hepaciviruses [19,20].

PUUV belongs to the family *Hantaviridae* (order *Bunyavirales*) and shows a broad spatial distribution in Europe, ranging from northern and central Europe to the Balkans and Russia [17,21–23] (more details are given in Discussion). PUUV transmission is exclusively horizontal and can be direct or via aerosolized excreta of infected voles. Viral RNA appears within 8–84 days post infection (dpi) in animals’ saliva, 11–44 dpi in feces, and 14–44 dpi in urine; the peak level for these excreta is reached between 11–28 dpi [24]. Outside the host, PUUV remains infectious for up to 12–15 days at room temperature but loses infectivity faster at increased temperatures: at 37˚C within 24 h, and at 56°C within 15 min in wet cell culture, but within a few hours under dry conditions [25]. Infected voles show PUUV-reactive antibodies in serum samples within 21 dpi or earlier [24,26,27]. Importantly, no vertical transmission from pregnant or lactating voles to their offspring was observed. This is because maternal antibodies (MatAb), which are transferred through the placenta and milk, temporarily protect vole offspring against PUUV infection [28]. The cited study also showed that offspring of infected mothers reared in a PUUV-contaminated environment did not develop their own antibodies until they were at least 80 days old. Thus, assuming that the time to develop antibodies is up to 20 days [24,26,27,29], the offspring may be protected from infection up to about 60 days of age, although this period may be shorter if antibody development takes longer in some individuals.

Humans can be infected with PUUV via direct contact with infected voles (e.g., by biting) or through inhalation of contaminated dust, but the virus is not transmitted between people. The infection can result in haemorrhagic fever with renal syndrome (HFRS), the severity of which is classified as mild to moderate, with symptoms usually similar to those of flu; however, 0.1–0.4% of cases were reported to be fatal [30]. Long-term monitoring studies indicated an association between increased numbers of human disease cases and both bank vole reservoir population abundance and PUUV prevalence in reservoir populations [31–34].

Among pathogenic infections in laboratory rodents, viral infections may have especially severe consequences due to zoonotic transmission to humans, even if they do not cause severe disease in the rodents. In the case of a viral infection that poses a danger to humans, the respective veterinary or sanitary authorities typically enforce extermination of the whole colony, as was done in the case of an outbreak of lymphocytic choriomeningitis virus (LCMV) in a house mouse (*Mus musculus*) colony in the USA [35]. This solution is well founded considering that (a) intravital detection of infected individuals before they spread the virus is hardly possible (antibodies in blood appear too late for that purpose [36]; (b) the virus cannot be eliminated by any known pharmacological treatment; and (c) although the immune system can often control the virus and the animals may not show obvious symptoms of disease, they chronically shed infectious virus, as is the case of bank voles infected with PUUV [36].

As the extermination of unique strains of laboratory animals is always a severe blow, alternative solutions are valuable. Because MatAb protect embryos, embryo transfer (ET) to non-infected surrogates could be considered as one such alternative. Although ET is feasible in laboratory mice, effective procedures for ET in other rodents, such as the bank vole, are not developed yet. Furthermore, ET is applicable when eradication of a virus from a few families is sufficient for rederivation of an inbred strain [37], but is inefficient when two hundred or more families should be cleaned to preserve genetic heterogeneity in several genetically polymorphic lines. Another solution could be cross-fostering newborns to non-infected mothers [38–40], although the procedure is not always successful [39]. Cross-fostering of bank vole newborns is very effective [41], but again the efficiency of applying this procedure to a large population is doubtful, as another equally large non-infected laboratory colony should be available as a source of foster mothers. Moreover, transferring newborns from infected mothers carries a high risk of also transferring the virus, which would then infect healthy foster mothers.

Here we describe a successful direct eradication of PUUV from the 16 valuable lines of bank voles [5,6] without the need for ET or cross-fostering. The procedure was based on the assumption that MatAb would protect newborn and juvenile voles from infection long enough to eliminate all infective PUUV sources from the environment. Thus, this successful eradication confirms the high efficiency of MatAb in protection of offspring from PUUV infection. As the pattern of infectivity and MatAb protection is similar to that of other hantaviruses [38], we believe the same approach could have a much wider application.

## Results

### Detection of PUUV and identification of the virus origin

The colony of bank voles was tested for the presence of PUUV-reactive antibodies using an enzyme-linked immunosorbent assay (ELISA) in 2008, and the result was negative. However, in December 2012, serological analyses using two assays (an in-house IgG ELISA and a commercial immunochromatographic Rapid test (ReaScan Ab-Dect Puumala IgG test, Reagena, Toivala, Finland; [42]) performed on 17 old individuals (aged 203–265 days) from generation 14 of the selection experiment showed that all of them were positive for PUUV-reactive IgG antibodies (S1 Table). In contrast, five bank voles from a different colony housed in separate rooms within the same facility tested negative for the antibodies. Subsequent RT-PCR targeting the small (S) segment, using RNA derived from lung tissue, confirmed infection in all 17 seropositive individuals (S1 Table). The pairwise nucleotide sequence similarities ranged between 99.1% and 100% (S2 Table). Similar levels of pairwise nucleotide sequence similarity were observed for the corresponding medium (M) and large (L) segment sequences (S2 Table).

Between August 2009 and March 2011, wild-trapped bank voles from nine populations, along with their offspring, were housed in the same facility for a separate project (see Materials and Methods). They were therefore considered a potential source of PUUV in the main colony. To identify the origin of the PUUV incursion, a phylogenetic analysis of the colony-derived PUUV sequences together with reference sequences of PUUV strains from various clades was performed. The results showed that the colony-derived sequences clustered with those of viruses detected in bank voles captured in Teleśnica Oszwarowa in southern Poland, one of the nine populations from which the live-trapped voles originated [43,44]. These sequences were clearly separated from those from Mikołajki in northern Poland [43,44] and from other sequences of the Russian clade (S1A–C Fig). Pairwise comparisons of partial S, M, and L segment sequences showed high similarity to sequences from Teleśnica Oszwarowa (S segment 98.0–99.8%; M segment 98.5–99.8%; L segment 99.2–99.7%), but much lower similarity to sequences from Mikołajki (S segment 82.8–83.5%; M segment 80.4–80.5%; L segment 86.6–86.8%) at the nucleic acid level (S2 Table). These results indicated that wild voles captured in Teleśnica Oszwarowa and temporarily held in the same facility were the source of the virus (see Materials and Methods).

Serological tests performed on humans after the virus had been detected in voles showed that all individuals who worked directly with the infected bank voles, as well as technicians who had changed the air filters in the ventilation system, had anti-PUUV IgG antibodies and thus had developed immunity against PUUV. Importantly, however, all five tested bank voles representing another colony maintained in the same facility were not infected, even though they were housed in rooms not separated by a sanitary barrier from those of the infected colony (S1 Table). Similarly, people who worked only with voles from the other colony, or worked in the facility without direct contact with the voles, as well as people who worked in the same building but outside the facility, were not positive for PUUV-specific antibodies. These results implied that the temporary maintenance of the colony did not pose an immediate risk to people who had already worked with the infected colony and had developed immunity [45], or to people working in the same building without contact with infected voles or contaminated dust. This was the basis for the decision of the sanitary authorities not to exterminate the colony immediately, but granting time for the attempted eradication of the virus (initially 6 months, later extended to 10 months).

### Outline of the eradication program

**Fig 1:**
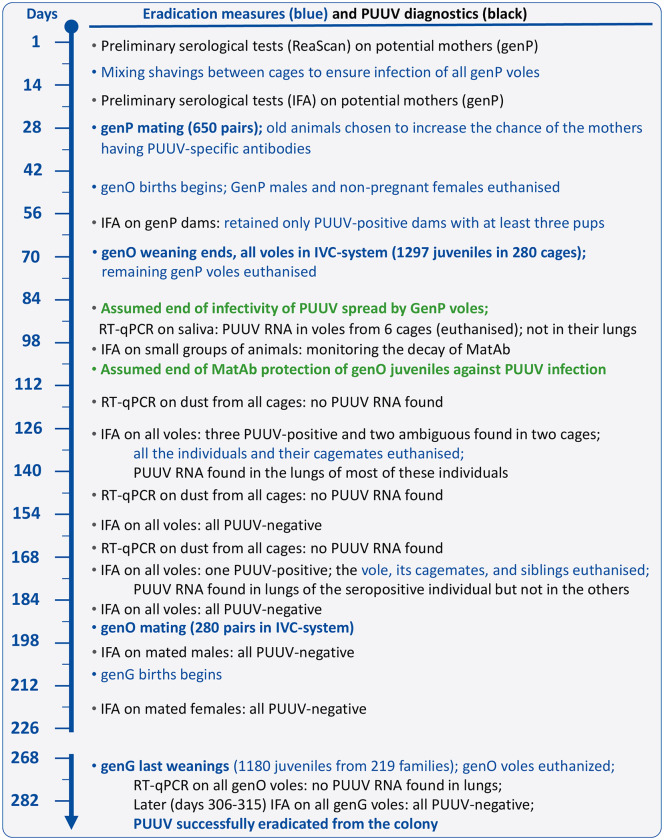
Timeline of Puumala virus eradication. The timing and range of the “safety window” set by the end of genP-produced PUUV infectivity and the end of post-weaning protection by MatAb (green text) was estimated based on [25] and [28]. Abbreviations: genP: parental generation, genO: offspring generation, genG: grand-offspring generation, IVC: individually ventilated cages, IFA: indirect fluorescent antibody assay, MatAb: maternal antibodies, RT-qPCR: quantitative reverse transcription-polymerase chain reaction, PUUV: Puumala hantavirus.

The plan for PUUV eradication (Fig 1) was founded on two previously mentioned reports: (a) outside the host organism, PUUV loses its infectivity at room temperature within a maximum of about 15 days [25], whereas (b) offspring of infected mothers are protected by MatAb, presumably at least until 60 days of age [28]. The voles in our colony are weaned at the age of 17 days. Thus, virus transferred from the maternal cage on the bodies or fur of juveniles is likely to remain infectious only until the voles are 32 days old (17 days up to weaning plus 15 days post-weaning). This gives, optimistically, a “safety time window” of 28 days (60 minus 32 days), or longer, before protection by MatAb becomes ineffective. Therefore, theoretically, if all voles from the parental generation (genP) are reproduced at the same time and euthanized immediately after weaning their offspring on day 17, the virus should not be effectively transmitted to the offspring generation (genO).

This apparently simple plan, however, was based on assumptions that could have been overly optimistic. Moreover, it posed several technical problems, particularly challenging when one considers the scale and conditions under which the work was performed. First, in order to preserve genetic variation in the 16 lines, we aimed to eradicate the virus from the offspring of at least 240 families, i.e., more than 1000 individuals. Second, all the work had to be performed in a facility that did not guarantee full protection against virus transfer between cages. These issues will be addressed in the Discussion.

The major problem was that the plan had to assume that some offspring would not be fully protected by MatAb and would become infected. Therefore, the offspring had to be kept in individually ventilated cages (IVC), ideally of a type warranting full protection against virus transmission, which was far beyond the available financial resources. Instead, the weaned voles were placed in AERO Mouse Green Line IVC (Tecniplast, Bugugiatte, Italy), which, although equipped with HEPA filters, were not designed to warrant full protection against virus transmission. Next, the animals and their environment had to be frequently tested to detect and eliminate infected individuals before they could infect others. However, negative test results suggesting the absence of virus-specific antibodies or RNA in samples that could be collected *intra vitam* (faeces, blood) do not provide firm evidence of virus absence. Definitive evidence can be obtained only by *post mortem* analyses. Therefore, to the simple “one-step” plan outlined above, we added another safety step. After confirming that none of the adult genO voles were seropositive, they were mated to produce another generation (grand-offspring: genG). After weaning genG, all genO individuals were euthanized and tested for the presence of PUUV RNA in lung samples. Finally, tests for the presence of PUUV were also performed on the next two generations.

### The eradication programme implementation

#### Procedures on parental generation (genP).

**Fig 2:**
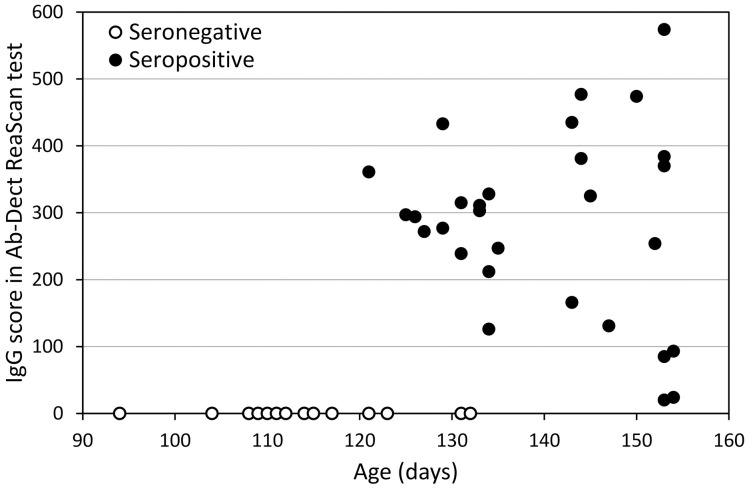
Relationship between anti-PUUV IgG antibody level measured as a score in the ReaScan Ab-Dect test and age of the voles. The results were obtained for 45 females from the parental generation (genP) tested to assess the age at which most animals become seropositive. The threshold values are: negative < 5, ambiguous (not observed), positive > 15.

The eradication programme started on 1 February 2013 (day 1; Fig 1). Preliminary tests for the presence of PUUV-reactive IgG antibodies (ReaScan Ab-Dect test), performed on 45 females at the onset of the programme, indicated that none of the 12 individuals aged 94–117 days was seropositive, 8 of 12 individuals aged 121–132 days were seropositive, and all individuals older than 132 days were seropositive (Fig 2, S3 Table). The results indicated that MatAb can provide protection for at least as long as reported in previous studies. However, to ensure that the potential mothers can transmit MatAb to their offspring, mating should be better postponed until all potential mothers are seropositive. It is also worth noting that, above the age of 132 days, the level of antibodies was not significantly correlated with age (Pearson r = -0.08, p = 0.73, n = 21), and that among the oldest individuals both very high and low values were observed (just above the “positive” threshold of 15; Fig 2).

To ensure that all females were infected, used bedding was mixed between cages (4, 3, and 2 weeks before the planned mating). Another series of preliminary tests (indirect fluorescent antibody assay, IFA), performed on days 19–20, showed that 12 of 28 individuals aged 108–119 days were seropositive, and only 9 of 25 individuals aged 121–126 days were positive. Thus, the results of both preliminary tests indicated that, to ensure that most of the potential mothers had developed anti-PUUV IgG antibodies, the females should be mated at an age of at least 130 days. Ideally, mating should be postponed even further, but the health and safety authorities only permitted us to keep the infected animals for a strictly limited time. Furthermore, all females giving birth must be tested for the presence of anti-PUUV antibodies before their offspring are included in the next steps of the programme.

On day 28 (Fig 1), 620 pairs were mated (35–40 per each of the 16 lines), and all remaining voles were euthanized. To increase the chance that only anti-PUUV IgG antibody-positive females were mated, and also to increase reproductive success, older individuals were chosen wherever possible (130–181 days old), but to ensure adequate representation in each of the lines, 25 females aged 117–128 days were also included. Wherever possible, the regular selection criteria used in the selection experiment were also considered [5]. Males were euthanized 12–13 days after mating (days 40–41). From day 17 after mating, females were checked daily for pregnancy and birth, and non-pregnant females were euthanized. The first births occurred 18 days post mating, and within 11 days 473 females gave birth (mostly in the first 6 days), while the remaining females were euthanized. On days 9–12 after parturition (days 54–63, Fig 1), cages were changed and IFA tests were performed on all 436 females that had reproduced and still had live offspring (S3 Table). Of the 18 females that had been mated at 117–127 days of age, only one tested negative for anti-PUUV IgG antibodies. Among the remaining 418 females that had been mated at 130 days or older, only three tested negative (mated at 131, 138, and 150 days of age). Further, we retained only mothers that (a) were seropositive, (b) gave birth up to 26 days post mating (to narrow the age span in the cohort of offspring to 9 days), and (c) had litters of 3 or more young. From those that met these criteria, we chose 280 (17–18 per line; 9 were mated below 130 days of age), whose offspring were weaned to IVC at the age of 17 days (days 63–71, Fig 1). Immediately after weaning, all genP voles and excess offspring were euthanized.

With the progressive reduction of the colony, all animal rooms and common work areas in the facility were carefully disinfected, including the ventilation system. From day 71 of the eradication programme, all potentially infectious material was confined to the IVC system, placed in a previously disinfected room equipped with an entirely independent ventilation system. All further work with the animals was also performed in this room. Tests for the presence of PUUV RNA in dust samples taken from various locations in the facility were performed repeatedly at a later date and, with a few exceptions, gave negative results. Results of these tests are detailed in the last section of the Results.

#### Procedures on offspring generation (genO).

The whole weaned litters were immediately placed in individually ventilated cages (IVC) equipped with HEPA filters (model G500, Aero “Green Line” system, Tecniplast, Bugugiatte, Italy), operating at 10% under-pressure and an air flow rate of 75 volume exchanges per hour. As the priority was to preserve the genetic pool, and hence to keep the offspring from as many families as possible, litters were reduced to 6 pups so that each family could occupy only one of the 280 available IVC.

According to [25], MatAb are expected to protect juveniles up to 60 days of age. However, because the last genP animals were euthanized when juveniles from the first litters were 25 days old, these juveniles remained protected for only 35 days after the last active sources of PUUV had been eliminated (day 106, Fig 1). In an attempt to reduce the duration of PUUV infectivity in the environment, the room temperature was set to 26°C instead of the usual 20°C for the first two weeks [25], until the end of the first round of cage and bedding changes. To prevent within-sibling mating, the litters had to be sexed before the voles reached sexual maturity. However, because of the limited number of IVC, same-sex individuals from different families had to be joined (up to six individuals from two families). This increased the risk of transmitting the virus from an infected family to others and also increased the risk of losing animals, especially males, as a result of inter-individual aggression. Therefore, to delay sexual maturation, already after weaning the photoperiod was changed to a short day (9L:13D) [13]. Separation of sexes was performed at the age of 28–39 days (days 84–85 of the eradication programme).

In an attempt to detect infection as early as possible, saliva samples were collected 11–12 days after weaning (days 77–86) from voles housed in 240 cages (pooled samples from all animals within each cage) and analysed using quantitative reverse transcription PCR (RT-qPCR). Viral RNA was detected in samples from six cages (S3 Table), and all 30 individuals from these cages were euthanized. However, a subsequent analysis of RNA extracted from their lungs indicated that the animals were not infected. Thus, the positive signal was probably due to contamination of the saliva sample with dust from fur, which could still contain the virus (or inactive viral RNA) transferred from maternal cages. Considering the difficulty of avoiding such false positive signals, we resigned from further attempts to analyse saliva samples. After two cycles of cage changes, on days 116–117, the first of three series of analyses for the presence of PUUV RNA in dust samples from the IVC was performed (S3 Table). All results were negative, with a few exceptions described below.

Between days 82 and 117, i.e., at the age of 36–71 days, six series of IFA tests were performed on small groups of animals (9, 17, 26, 14, 15, and 24 individuals, partly repeated) to monitor the loss of PUUV-specific MatAb. Among 17 individuals aged 36–42 days, MatAb were present in 13 individuals, absent in one, and in three the result was ambiguous (see Materials and Methods for a description of the scoring scheme). At the age of 49–50 days, 12 individuals were positive, 9 negative, and 4 uncertain, and at the age of 56–64 days only 3 were positive, 12 were negative, and 14 uncertain. Of 24 individuals tested at the age of 71 days (all for the first time), only 1 individual was positive, 16 were negative, and 5 uncertain. Therefore, ‘positive’ results from tests performed on animals aged about 75 days or more should be considered highly indicative of PUUV infection.

On days 130–139 (i.e., at 79 – 93 days of age), the first series of IFA tests were performed on all individuals to detect the presence of anti-PUUV IgG antibodies and identify infected voles (S3 Table). The test was positive for three individuals from two families, maintained in two separate cages. The mothers of those families tested positive for anti-PUUV IgG antibodies on day 10 after parturition, but did not have the antibodies 10 days before mating. They were mated at 117 and 125 days of age, i.e., below the threshold of 130 days, above which all individuals in the parental generation were expected to test positive for anti-PUUV IgG antibodies (as explained in the previous section). In addition, two voles from one of the two cages had ambiguous test results (one from the same family and one from a different mother, who was mated at 179 days of age). All these individuals and their cagemates were euthanized on day 134. Analyses of lung tissue confirmed the presence of viral RNA in all seropositive individuals, as well as in two of their four cagemates. Viral RNA was also found in dust taken from these cages. However, the IFA tests were negative in their siblings maintained in separate cages. The results remained negative in three subsequent tests, including a test performed on day 166, i.e., 82 days after separating the siblings. However, a subsequent test, performed on day 175, revealed a positive result in a single individual (aged 131 days), a sibling of one of the infected individuals detected earlier (euthanized on day 135). Therefore, the single sibling of the individual newly detected to be positive (kept in the same cage) and the remaining siblings of the previously infected animals (kept in separate cages) were also euthanized, even though they were PUUV-negative. Tests for the presence of PUUV RNA in lung tissues showed that the seropositive individual was indeed infected, but all other individuals were not.

#### Rearing the grand-offspring generation (genG).

Another series of IFA tests, performed on days 182–187 (Fig 1; S3 Table), revealed no seropositive individuals, and no viral RNA was detected in dust samples from cages. However, the previous observation of a single infected individual, in which antibodies were not detectable 82 days after the last contact with an infected individual but were detected on day 92 after the last contact, showed that a negative IFA result could not be regarded as proof that voles do not carry the virus. Tests for the presence of viral RNA in lung samples can provide such evidence but require sacrificing the animals. Therefore, the next, grand-offspring (genG) generation had to be produced under controlled conditions before eradication could be confirmed.

Therefore, the photoperiod was reset to a long day (16L: 8D), and the voles were mated again on day 190 (Fig 1). As the entire procedure had to be performed in the IVC system, the number of pairs was limited to 280. The mated individuals were chosen to maximize representation of independent families within each line. All genO voles were again tested for the presence of PUUV-specific antibodies, and the results were negative. All reproductive females were euthanized after weaning the offspring, and all other genO individuals were euthanized earlier. No PUUV RNA was found in their lung tissues (S3 Table).

Thus, as voles from the offspring generation were not infected and the environment was also free of infectious PUUV (see below), young voles from the grand-offspring generation (1180 individuals from 219 families) could also be declared PUUV-free. Eradication was confirmed by negative IFA tests for PUUV-specific antibodies performed at the age of 59–100 days in all 1166 animals that survived to this age, and by negative RT-qPCR tests for RNA presence in lung tissue sampled from 38 adult individuals (aged 126–164 days).

The successful eradication was further confirmed by tests performed in the subsequent two generations (S3 Table). In the first of them (genG + 1), RT-qPCR showed no PUUV RNA in lung samples from 50 adult individuals (aged 113–178 days). In the next one (genG + 2), IFA tests showed no PUUV-specific IgG antibodies in blood samples from 97 individuals (aged about 140 – 240 days), and RT-qPCR tests showed no PUUV RNA in lung tissue samples from 21 individuals (aged 185–228 days).

#### Tests for the presence of PUUV RNA in the facility environment.

Parallel to the procedures performed on voles, we repeatedly conducted RT-qPCR assays to detect PUUV RNA in dust samples collected from various locations within the facility (S3 Table). The first sampling was performed on day 89 (Fig 1), when all voles had already been confined to the IVC system and housed in a single room, while the remaining rooms and common areas (corridor, cage wash room) had been cleaned and disinfected. Of the seven samples collected from the central corridor and cage wash room, one tested positive for PUUV RNA. The area was subsequently re-cleaned and disinfected with Virkon (LANXESS, Sudbury), and none of the seven samples collected the following day tested positive in the RT-qPCR.

On days 147 and 162, a total of 17 dust samples were collected from five freezers and from exhaust ducts of the IVC system. PUUV RNA was detected in one freezer and in an exhaust duct from the IVC section ventilating cages that had housed the last few PUUV-positive voles. The contaminated freezer was disinfected and the filters were replaced; however, cleaning of the entire IVC system had to be postponed until completion of the eradication procedure.

The next series of tests of dust samples collected from multiple locations within the facility was conducted at the conclusion of the eradication procedure (30 samples collected on days 264–270) and repeated 2–4 weeks later (53 samples collected on days 285–299). No PUUV RNA was detected in the most critical locations, including IVC system filters, the bedding disposal station, the central corridor, the cage wash room, freezers, and the central ventilation system. However, PUUV RNA was detected in four samples collected from ventilation ducts in three rooms. Given that these samples were collected more than 6 months after all potentially infected voles had been confined to the IVC system, and more than 3 months after the last infected vole maintained in the IVC system had been euthanized, we infer that the RT-qPCR assay detected either non-infectious virus or residual RNA fragments originating from the period when the entire colony was infected, rather than infectious PUUV. Nevertheless, all affected areas were cleaned and disinfected again.

## Discussion

### Identification of the PUUV origin in the bank vole colony

Multiple molecular investigations have indicated the evolution of PUUV within local bank vole reservoir populations, which is reflected in the close phylogenetic relationship of PUUV sequences from bank voles from the same site [46,47]. The current distribution of PUUV lineages is mainly shaped by the postglacial immigration of bank vole phylogroups [48]. Thus, central and western Europe were invaded by bank voles of the western phylogroup, distributing the central European (CE) PUUV lineage [49]. In contrast, in eastern and northern Europe the situation is much more complex. Different PUUV lineages can be harboured by different phylogroups of the bank vole. So far, in northern Poland exclusively the Latvian (LAT) PUUV lineage has been detected in bank voles of the Carpathian and Eastern phylogroups, whereas in southern Poland PUUV of the Russian (RUS) lineage has been demonstrated to be harboured by bank voles of the Carpathian and Eastern phylogroups [43,44].

The PUUV sequences of the 17 bank voles from generation 14 of the selection experiment (i.e., the grandparent generation in terms of the eradication programme) were almost identical, which suggests a single incursion event. To identify the potential origin of the PUUV incursion, these colony-derived sequences were compared to reference sequences of PUUV strains from various clades, indicating a high nucleotide sequence similarity and phylogenetic clustering with sequences from Teleśnica Oszwarowa, southern Poland. Therefore, the infection probably originated from the group of voles that were trapped in Teleśnica Oszwarowa and temporarily maintained (2009–2011) in the same facility as the selection experiment colony. Based on the eradication experiment described here, future analyses will allow determination of the mutation rate of PUUV in bank voles and comparison with the situation in cell culture [46].

### The eradication scheme and its pitfalls

The simple plan for PUUV eradication outlined in the second section of the Results was based on two assumptions supported by fairly convincing empirical data: (a) PUUV loses its infectivity outside the host at room temperature within about 15 days [25], and (b) the offspring of infected mothers are protected by MatAb until the age of 60 days or longer [28], and on a simple calculation leading to the conclusion that if young voles are weaned and separated from any infected mother at the age of 17 days, the virus transmitted on their bodies will become non-infectious approximately 4 weeks before protection by MatAb ceases. Nevertheless, the plan was vulnerable to many potential problems, which should be considered by other researchers who may need to adopt this scheme in their work:

1) The estimates of both the MatAb protection period and the PUUV infectivity duration in the environment were based on small or only moderately sized samples. Thus, neither the minimum of the former nor the maximum of the latter could be determined with high reliability. If the actual extremes deviated from the reported values by just 10 days, the optimistic 4-week safety window would become very narrow.
2) The safety window considered for the entire colony is in practice much shorter because, even if all parents are mated at the same time, parturitions are spread over several days, which again narrows the safety window (in our case to 20 days).
3) The plan requires that all potential mothers are infected well before mating and have developed a sufficiently high level of PUUV-specific antibodies to provide their young with effective protection by MatAb. To help achieve this, we used a paradoxical approach of intentionally infecting all animals before implementing the proper eradication procedure. However, the available quantitative antibody-detection tests require a large amount of blood and practically cannot be performed without euthanizing the animals. On the other hand, the available rapid immunoassays require only a drop of blood but provide only qualitative or semi-quantitative results. To our knowledge, no studies have been performed to determine whether and to what extent the efficiency of MatAb-mediated protection in newborns and juveniles depends on the level of anti-PUUV antibodies in the mothers. Thus, a mother “positive” according to such a qualitative or semi-quantitative test might not provide effective protection to the offspring.
4) Moreover, we had to choose between three conflicting requirements: (a) maintaining a balanced representation of all 16 vole lines; (b) mating females at an age at which they were highly likely to have already developed antibodies; and (c) the understandable requirement of the health inspection to begin and complete the eradication process as quickly as possible. Therefore, in the case of lines with a small number of older females, we also mated younger females (aged 117–127 days) and retained the offspring of nine of these mothers. Although the IFA test showed the presence of anti-PUUV IgG antibodies in all of these mothers around 10 days after parturition, two of the younger mothers were the only ones that had inadequately protected their offspring against PUUV infection.
5) Importantly, even a single incompletely protected juvenile would cause re-occurrence of the infection in the next generation unless the individual is isolated and eliminated before transmitting the virus. However, the virus starts spreading before PUUV-specific antibodies in blood can be detected [24]. Moreover, serological assays do not allow distinction between a young individual still carrying MatAb and one that has started to produce its own antibodies as a result of infection. Tests for the presence of viral RNA in saliva or excreta may provide positive evidence of infection but do not provide negative evidence (that the animal is not infected), because virus shedding is discontinuous [24]. Samples from the cage environment could be a more effective measure, but if applied soon after weaning they would result in false positives, because some amount of virus or viral RNA may be carried from maternal cages on the juveniles’ bodies. To summarize, no *intra vitam* method allows reliable detection of infection before the virus starts spreading, and in practice not even at the time when spreading already occurs.
6) Thus, the eradication plan must assume that some young individuals may be infected. Therefore, all young animals must be kept in isolation, which requires the use of IVC and an appropriate cage-changing station to prevent virus transmission during animal maintenance procedures and blood testing. Such equipment, at the level of protection designed for preventing viral transmission (grade III), and with sufficient capacity to maintain several hundred individuals, was far beyond our financial reach, and the same may be true for most other researchers. We could afford to purchase only a grade II + IVC system (“Aero Green line”, Tecniplast, Italy), which, although equipped with HEPA filters, is not guaranteed to prevent viral transmission. In our case, the cage system and work scheme that we implemented proved to be effective: the few infected animals found in two cages did not spread the virus to other voles. However, this should not be regarded as strong evidence that such a system provides adequate protection in general.
7) We initially assumed that the absence of PUUV-specific antibodies after a 55-day quarantine (a maximum of 15 days of PUUV infectivity outside the host plus a maximum of 40 days for developing own antibodies after infection) could be treated as evidence of successful eradication. However, the case of a single vole that did not show antibodies in the test conducted 82 days after its last contact with an infected vole or infectious material, yet was detected as seropositive on day 92 (at 131 days of age), changed this perspective. Regardless of the mechanism of delayed seroconversion, which remains unknown, or a false negative result in the initial testing, we concluded that no period could be considered long enough to provide definitive negative evidence. Therefore, the one-step eradication plan has no predefined moment at which success or failure can be declared with certainty.
8) Negative evidence can be provided only by *post mortem* tests for the presence of viral RNA in lung tissue (or perhaps other organs), and by tests for the presence of viral RNA in the environment, which have proved to be extremely sensitive and allowed detection in dust samples for at least 3 months after infectious virus material was removed. Therefore, the eradication programme must *a priori* include the second step: producing, within the IVC system, a next generation of offspring and performing RNA analyses in the lung samples of all parents (or in another organ in which a particular virus resides).
9) This raises the question of whether it would be better to schedule the second reproduction as early as possible, i.e., just after the animals reach sexual maturity. In the case of bank voles, this would be at around 60 days of age, which would considerably shorten the whole procedure. However, we would not recommend this because of the increased risk that some individuals are infected even though not yet detected as anti-PUUV-positive by serological testing, and hence an increased risk of transmitting the virus to the grand-offspring generation.
10) This was not a planned experiment, but rather a “rescue operation” carried out on a tight budget and with a “fast-track design”, i.e., involving instant decisions about subsequent steps based on current information. Consequently, not all procedures were well optimised and some decisions were imprudent. Therefore, if we could, we would introduce a few changes to the procedure:

First, we would not have mated parental generation females that had not tested positive for the presence of anti-PUUV IgG antibodies before mating. Including in the programme the few younger females that did not have antibodies 10 days before mating was perhaps the only major fault. If we had not done so, we probably would not have had to cope with infected individuals in the offspring generation. Thus, we would have argued to the health and sanitary authorities for a further delay in starting the eradication programme. If permission had not been granted, we would have sacrificed the balanced representation of all vole lines. Also, if we could afford that, it would have been better to test all females before mating and during lactation with the more reliable semi-quantitative test (ReaScan Ab-Dect), rather than the much less costly qualitative IFA test. The variation in antibody levels was presumably very high (Fig 2), and such tests would have allowed selection, for further steps, only of offspring from females with high antibody levels. Similarly, all weaned young could have been individually tested with the semi-quantitative test for the level of MatAb, and only those with high levels retained. Possibly, in this way we would have avoided having incompletely protected young in the offspring generation, and the entire procedure would have been shortened.

Second, we would not attempt to detect viral RNA in the saliva of young animals 12 days after weaning. This is because contamination of samples with virus transmitted at weaning, which is still present in the environment, leads to “false alarms” and unnecessary euthanizing of animals. In fact, it is not worth investing in the tedious attempts to sample saliva, which inevitably also increases the risk of transmitting the virus between cages. On the other hand, sampling dust from cages is easy, provides integrated information about virus shedding over several days, and such tests have proven to be highly sensitive. Again, however, such tests should only be performed after a longer period since weaning and after at least two rounds of cage changes.

Third, after detecting the first few infected individuals about 50 days after weaning, we eliminated only their cagemates, but not their sibling or the cagemates of those siblings, which were not seropositive. We did this because, at that time, we did not know how many of the young animals were infected. After losing many animals due to a “false alarm”, we were reluctant to eliminate more without evidence of infection. Instead, the suspicious animals were tested more frequently. However, from the perspective of later experience—detecting the infected sibling more than 40 days later—we see that this decision was wrong, and that all those suspicious animals should have been eliminated as well.

## Conclusion

In the presented case, we demonstrated that, once the pattern of pathogen infectivity is understood, it is possible to eradicate viral contamination from an animal facility by implementing improved logistics and moderately advanced safety precautions. This approach enables the valuable colony to be maintained without drastically reducing population size (compared to other methods such as ET or cross-fostering), thus preserving its genetic polymorphism. Given the scientific and financial benefits of maintaining the animal model system, our procedure could be considered an alternative to complete liquidation of the population in similar cases.

When implementing the rescue procedure, based on a very large number of individuals, we confirmed two facts about PUUV reported by other authors [28]: (1) it is not transferred via the placenta, and (2) MatAb are transferred via breast milk from mothers and, for a certain time, protect juveniles from infection. The same appears to apply to other hantaviruses. For example, in young rats armed with MatAb and intentionally infected with Seoul orthohantavirus (SEOV; species *Orthohantavirus seoulense*), the peak of anti-SEOV-IgM antibody formation appears about 2–3 months post infection, whereas in non-MatAb-protected animals it appears already about 2–3 weeks post infection [38]. Therefore, we believe that the eradication scheme we have conceived and implemented to eradicate PUUV from the bank vole colony can also be effective in the case of other hantaviruses, and perhaps also other viruses infecting other animal species, provided that the two main characteristics—protection of embryos and protection of juveniles by MatAb—are similarly effective.

## Materials and methods

### Ethical statement

Animal welfare was monitored daily throughout the experiment. The procedures on animals performed as a part of the maintenance of the experimental colony were approved by the 1st Local Institutional Animal Care and Use Committee in Kraków (decision 68/2012), in accordance with the EU directive 2010/63/EU. According the regulations effective in 2013, the additional procedure of blood sampling performed exclusively for the purpose of the PUUV detection, rather than as a part of an experiment performed for research purposes, did not require a separate ethical permit.

### Establishing the colony and the selection experiment

The colony of bank voles (*Myodes glareolus* syn. *Clethrionomys glareolus*) was established from wild voles captured in Niepołomice Forest near Kraków (southern Poland) in 2000 and 2001 [50,51]. In 2004, a multidirectional artificial selection experiment was initiated, with 4 lines selected for high swim-induced aerobic metabolism, 4 for herbivorous capability, 4 for predatory propensity, and 4 unselected control lines [5,6]. To preserve genetic polymorphism, in each of the 16 lines the offspring were reared from 16–20 families in each generation (two per year). The selection was effective in each of the three directions, and the unique experimental evolution model system has become a valuable resource for research in several areas, such as behaviour [52,53], metabolism and thermoregulation [6,54,55], molecular genetics [56,57], exercise physiology [58,59], neurophysiology [60,61], ageing [62–64], ecotoxicology [65], stress response [66,67], holobiont evolution [41,68] or evolution of dietary niche [69].

Details of the breeding scheme and maintenance conditions are described in our earlier work [6,52]. Briefly, the colony has been maintained in a “conventional” facility, corresponding to Biosafety Level 1, appropriate for the intended research objectives, which assumed that the animals should not be maintained under germ-free conditions. Depending on the phase of breeding, the colony size ranges from about 1500–4000 individuals. The animals are kept in standard plastic mouse cages (1290D or 1264C, Tecniplast, Bugugiatte, Italy) with sawdust bedding, located in six climatic chambers providing fixed light conditions (16L:8D), a constant temperature (20°C), and appropriate ventilation. Food (Labofed H, Morawski, Kcynia, Poland) and water are available *ad libitum*.

### Other voles in the facility

In the same facility, another permanent but small bank vole colony is maintained, established in the 1970s, which forms the basis of a separate research programme by our colleagues [70]. The animals are maintained in a partly separate space, which includes animal rooms and laboratory space. However, this space is not separated from the area where the voles from the selection experiment are kept by a sanitary barrier: it is connected to the common corridor by a standard door and uses the same washing and food storage rooms. Voles from this colony have not been infected by PUUV.

Between August and November 2009, a separate temporary colony of voles was established based on animals captured from nine wild populations (between 33 and 74 adults and subadults per population). This colony was used to study variation in physiological performance traits [71,72]. The animals were kept in a separate room in the same facility as the main colony. The maintenance conditions in this temporary colony were the same as in the main colony. However, the working regime was separate (people did not work in both colonies on the same day, and separate sets of cages, bottles, and other equipment were used). Again, however, the room was not separated by a strict sanitary barrier. Then, two generations of offspring of the wild animals were produced, and finally the entire colony was closed in March 2011. The wild-derived animals were present in the facility from the time when animals of generation 8 of the selection experiment were bred until the time when animals of generation 11 were bred.

### Anaesthesia, euthanasia, and sample collection

For the purpose of the initial investigation, 22 voles (17 from the focal colony and 5 from the other colony maintained in the same facility) were anaesthetized with isoflurane (Aerane, Baxter, USA), and blood was collected by cardiac puncture using a heparinized syringe. Voles were then euthanized by administering increasing doses of isoflurane until cardiac arrest, dissected, and the heart and other internal organs were removed (which ensured death). The blood was centrifuged at 4000 rcf for 10 minutes and the plasma was separated. Plasma and samples of internal organs were stored at −80°C and carcasses at −20°C, and then transferred for further analyses performed later at the Friedrich-Loeffler-Institut.

For the purpose of the massive and repeated testing for the presence of PUUV-specific antibodies during the eradication procedure (see next section), 5–10 µl blood samples were taken from the femoral artery of live animals. Animals from the offspring generation (genO) that were detected or suspected to be infected with PUUV based on either the presence of anti-PUUV IgG antibodies or PUUV RNA in saliva, as well as their cagemates and siblings, were immediately euthanized by cervical dislocation. The euthanasia of large numbers of animals planned at the termination of subsequent generations was performed either by cervical dislocation or CO_2_ inhalation.

### Testing for PUUV presence during the eradication programme

#### Serological tests.

Because the eradication plan required repeated testing of the same animals, and obtaining results as soon as possible after sampling, we had to rely on quick, semi-quantitative or qualitative, low-cost tests that can be performed on a sample of 5–10 μl of blood.

The initial tests performed in genP animals, aimed at assessing the age at which all potential mothers could be expected to be seropositive, were carried out with a commercial immunochromatographic rapid test based on purified nucleocapsid protein of PUUV (ReaScan Ab-Dect Puumala IgG, Reagena, Toivala, Finland; [42]). Blood samples of 10 µl were taken from the femoral artery. The samples were mixed with the dilution buffer containing anti-mouse IgG-coated gold particles and transferred into the test cassette. There, anti-PUUV IgG antibodies are captured by the membrane-bound PUUV antigen. The ReaScan reader reports the concentration of PUUV-specific antibodies in a numerical, but unitless form. According to the manufacturer’s instruction, the results were scored as anti-PUUV-negative when below 5, positive when above 15, and ambiguous between 5 and 15.

As the whole eradication plan required performing several thousand tests, for financial reasons the main testing was performed with a low-cost classical indirect fluorescent antibody assay IFA test (Puumala virus IFA slide, HaartBio Ltd, Helsinki, Finland [73]), which provides only a qualitative assessment. The IFA tests were used to determine the presence of anti-PUUV antibodies for four purposes: (1) testing the dams from genP after parturition to retain only the offspring reared by PUUV-positive ones, (2) testing a group of young voles from genO to assess the age at which MatAb are lost, (3) recurrent mass testing of all individuals from genO (offspring) and genG (grand-offspring) for signs of PUUV infection, and (4) testing smaller groups of voles from the neighbouring colony maintained in the same facility to monitor their infection status.

The tests were performed on 5 µl of blood sampled from the femoral artery, diluted in sterile phosphate-buffered saline (PBS) and stored on ice or in a refrigerator until analysis (performed on the same day). Tests 1, 2, and 4 were performed individually, and the blood dilution was 1:10. For the mass tests (test 3), performed repeatedly on all animals (5 rounds), blood samples from cagemates were pooled after sampling to increase throughput and reduce costs. Typically, the dilutions ranged from 1:4 (when six voles shared a cage) to 1:10 (when only one individual was in the cage). Pilot tests showed that a pooled blood sample from one positive and five negative individuals allowed the sample to be correctly identified as positive. When a pooled sample tested positive, the test was repeated for individual voles.

The analyses were performed according to the manufacturer’s instructions. PUUV-specific antibodies present in PBS-diluted blood or positive control (bank vole sera) bind to the PUUV antigen presented in the slide wells after 30 min of incubation in a humid chamber at 37°C. Unbound antibodies were washed away with PBS and distilled water. Fluorescein isothiocyanate (FITC) conjugated to polyclonal rabbit anti-mouse IgG (product F0261, Dako, Glostrup, Denmark) was added to all wells to enable detection of antigen–antibody binding under a fluorescence light microscope (Eclipse 80i, Nikon Instruments Inc., Melville, NY, USA). The wells were evaluated immediately after completing slide processing.

Several preliminary trials with the IFA test were performed to establish a grading scale based on fluorescence brightness, and the scale was learned by all persons involved in testing. Initially, the scale had six grades (from 0 = “certainly negative” to 5 = “strongly positive”), but it was later reduced to four levels: 0 = certainly negative, 1 = ambiguous negative, 2 = ambiguous positive, and 3 = strongly positive. During the main work, each slide was evaluated independently by two observers. If both observers scored the sample as either positive or negative, the result was considered confirmed. Otherwise, the test was repeated on the next day using a new blood sample. Positive and negative controls for the IFA tests were mixed blood samples collected at the time of euthanasia (and stored at −80°C) from genP animals previously tested and classified with the ReaScan Ab-Dect test.

#### Viral RNA detection.

To detect infected individuals from genO during the eradication programme, samples of saliva from live animals and dust from their cages were collected for viral RNA detection. Saliva samples were collected as described in [24]. The mouth of each vole was swabbed using a separate sterile cotton swab. Immediately after sampling, swabs from all individuals in a cage were placed in a single cryotube containing 500 μl of dilution medium (Hanks’ balanced salt solution containing 2% HEPES, 2% fetal calf serum, and 1% penicillin–streptomycin [all compounds from Sigma-Aldrich, St. Louis, MO, USA]). Dust from cages was sampled in the same way, using sterile swabs. Up to four swabs were placed in one cryotube with the same buffer as used for saliva samples. The tubes with samples were stored at −80°C and processed within the following few days. To confirm infection in genO animals that tested positive for anti-PUUV antibodies in IFA or for presence of PUUV RNA in RT-qPCR on saliva samples, and to confirm the absence of PUUV at the final stage of the eradication programme, lung samples were collected for RNA analysis. Animals were euthanized (cervical dislocation or CO_2_ inhalation) and dissected, and lung samples were placed into cryotubes with RNAlater. Additionally, to confirm the effectiveness of facility disinfection, dust samples from various locations in the animal facility were collected and preserved as described above.

Detailed protocols of RNA extraction and analysis are provided in supplementary information (S1 Protocols). Briefly, RNA from all samples was extracted using RNAzol (Molecular Research Center, Cincinnati, OH, USA). At the first stage of extraction, lung tissue was homogenized using a motor homogenizer with diethyl pyrocarbonate (DEPC)-treated metal tips (2 × 15 s). RNA extracted from lung samples of four individuals was pooled and analysed as a single sample.

To detect PUUV RNA, quantitative reverse transcription PCR (RT-qPCR) was performed according to the QuantiTect Probe RT-PCR Kit protocol (Qiagen, Venlo, Netherlands), using primers F (5´-gtgcaccagatcgrtgtcc-3´) and R (5´-yarctctgccatccctgca-3´) and a TaqMan probe (5´-ccaacatgyatttatg-3´) (Syngen Biotech, Wrocław, Poland) (protocol of [24], modified according to personal advice from Liina Voutilainen). Amplification was performed with an automated 7500 Fast Real-Time PCR System (Applied Biosystems Inc., Foster City, CA, USA). The amplification programme consisted of one cycle of initial incubation at 50°C for 30 min and denaturation at 95°C for 15 min, followed by 45 cycles of annealing at 94°C for 15 s and extension at 59°C for 60 s [24]. Each set of analyses included a negative control (all reagents without RNA sample) and a positive control (all reagents with pooled RNA extracted from lung samples of several voles with known anti-PUUV IgG levels, identified at the beginning of the eradication procedure). Up to 86 samples were analysed in one run.

The instrument reported results as positive or negative based on the cycle number (Ct) at which the threshold level of the normalised reporter signal (ΔRn) exceeded a threshold set separately for each run (ranging from 0.019 to 0.039). If the threshold was not exceeded by the 45th cycle, the sample was scored as RNA-free (negative). Otherwise, it was scored as containing PUUV RNA. In positive control samples, as well as in samples in which the virus was detected, Ct values ranged from about 27–41. Final results for all samples were recorded in the database as either positive or negative.

### Identification of the putative PUUV origin

#### Serological investigations.

Chest cavity lavage (CCL) samples were analysed using the same commercial rapid test (ReaScan Ab-Dect Puumala IgG) and an in-house IgG ELISA as previously described [42,74]. As a positive control, the monoclonal antibody (mAb) 5E11 was used [75]. CCL from a bank vole previously tested negative by serological and molecular methods and originating from a region where PUUV is absent was used as a negative control.

#### Molecular investigations.

For identification of the origin of PUUV incursion, RNA was isolated from a lentil-sized piece of lung tissue (about 30 – 100 mg) from 22 bank voles using an in-house protocol and QIAzol Lysis Reagent (QIAGEN, Hilden, Germany) [76]. Conventional RT-PCR assays targeting the S, M, and L genome segments were applied as described previously [74,77,78] using the SuperScript III One-Step RT-PCR with Platinum TaqKit (Invitrogen, Darmstadt, Germany). The positive control was virus isolate-derived RNA, and RNase-free water served as the negative control. RT-PCR products of the expected size were sequenced by the dideoxy-chain termination method with the BigDye Terminator v1.1 Kit (Applied Biosystems, Darmstadt, Germany). Sequence alignments were established with the software BioEdit v7.2.5 as well as pairwise sequence comparison [79]. The most suitable substitution model was determined using jModelTest v2.1.8 [80]. Phylogentic trees were reconstructed using MrBayes v3.2.6 [81,82].

### Sanitary precautions

All work associated with standard animal maintenance, as well as collection of samples for PUUV testing (blood and tissue samples from voles, and dust samples from cages and the facility environment), was carried out by personnel who had been confirmed to possess anti-PUUV IgG antibodies and were therefore protected against reinfection with PUUV. Nevertheless, personnel wore anti-dust or medical masks, disposable gloves (usually latex gloves over veterinary gloves), disposable non-woven aprons with elasticated cuffs worn over medical aprons, and disposable non-woven shoe covers and caps. Personnel cleaning cages also wore waterproof aprons. After work, all personnel changed their clothing and disinfected their hands with Manusan (an antiviral, antibacterial, and antifungal liquid; Laboratoria Polfa Łódź, Warsaw). All work was performed under controlled conditions, minimising virus transmission. Manipulations requiring opening cages (i.e., changing cages, providing food, taking samples, removing used bedding) were performed using a cage-changing station and bedding disposal station with laminar airflow and HEPA filters (models CS5 and DS36, Tecniplast, Bugugiatte, Italy). All tools, equipment, working surfaces, chambers, corridors, and facility rooms (storage, cleaning, etc.) were continually disinfected with an antiviral agent (Virkon; LANXESS, Sudbury). The entire ventilation system in emptied rooms was disinfected soon after moving juveniles into the IVC system.

## Supporting information

S1 Fig Phylogenetic trees of partial Puumala virus (PUUV) sequences from the laboratory (lab. **) colony and reference sequences from Poland and other representative strains.** The sequences are from A) the S segment with 711 nucleotides (nt), B) M segment with 618nt, and C) L segment with 411nt in length. The consensus sequences and alignments were constructed with BioEdit v7.2.5 [83] and the best substitution model determined with JModelTest v2.1.8 [80]. The phylogenetic trees were calculated with the aid of MrBayes v3.2.6 with up to 4 x 10^6^ generations, a burn in of 25% and two-parameter substitution models with gamma distribution and invariant sites [81]. PUUV lineages: ALAD Alpe-Adrian, CE Central European, DAN Danish, FIN Finnish, LAT Latvian, N-SCA North-Scandinavian, RUS Russian, S-SCA South-Scandinavian. Outgroups: MUJV Muju virus, TULV Tula virus. [References are in the supplement].
(PDF)

S1 Table Serological and RT-PCR analyses of 22 bank voles of the generation 14 of the bank vole colony. (XLSX)

S2 Table Pairwise nucleotide (nt) and amino acid (aa) sequence similarities of partial S (a), M (b) and L (c) segment sequences of Puumala virus (PUUV) detected in the colony, reference sequences from Poland, and other representative strains of PUUV clades. (PDF)

S3 Table Summary of the serological and RT-qPCR tests performed during and after the eradication procedure. (XLSX)

S1 Protocols Protocols of the RNA analyses performed during the PUUV eradication program. (PDF)

S1 Raw images Raw images of agarose gels from the RT-PCR analyses targeting the segment S of PUUV. (PDF)
