# Supplementary material for: Maternal antibody-mediated elimination of a Puumala hantavirus outbreak in a bank vole colony
Source: PLoS Pathog. 2026 May 29;22(5):e1013693. doi: 10.1371/journal.ppat.1013693 (PMC13241010; doi:10.1371/journal.ppat.1013693)
Supplement: S2 Table — (PDF) [file ppat.1013693.s003.pdf]

S2 Table. Pairwise nucleotide (nt) and amino acid (aa) sequence similarities of partial S (a: 711 nt length), M (b: 618 nt length) and L (c: 411 nt length) segment sequences of Puumala virus (PUUV) detected in generation 14 (gen14) of the colony, reference sequences from Poland, and other representative strains of PUUV clades.

(a) Reference sequences of the S segment	PV798420 1gen14 Kraków Poland (RUS)		PV798421 6gen14 Kraków Poland (RUS)		PV798422 9gen14 Kraków Poland (RUS)		PV798423 19gen14 Kraków Poland (RUS)	
	nt	aa	nt	aa	nt	aa	nt	aa
PV798420 1gen14 Kraków lab. colony Poland (RUS)	-	-	**0.998**	1	**0.998**	1	**0.998**	0.995
PV798421 6gen14 Kraków lab. colony Poland (RUS)	**0.998**	1	-	-	0.997	1	0.997	0.995
PV798422 9gen14 Kraków lab. colony Poland (RUS)	**0.998**	1	0.997	1	-	-	0.997	0.995
PV798423 19gen14 Kraków lab. colony Poland (RUS)	**0.998**	0.995	0.997	0.995	0.997	0.995	-	-
KX815395 KS14/118 Teleśnica Oszwarowa Poland (RUS)	0.987	0.991	0.985	0.991	0.985	0.991	0.985	0.987
KX815396 KS14/121 Teleśnica Oszwarowa Poland (RUS)	**0.998**	0.995	0.997	0.995	0.997	0.995	0.997	0.991
KX815394 KS13/855 Mikołajki Poland (LAT)	0.835	0.978	0.834	0.978	0.834	0.978	0.835	0.974
JN657228 Latvia (LAT)	0.838	0.978	0.839	0.978	0.836	0.978	0.838	0.974
JN657230 Latvia (RUS), JN657231, JN657232	0.872-0.876	0.974	0.870-0.874	0.974	0.870-0.874	0.974	0.870-0.874	0.970
AJ314598 Estonia (RUS), AJ314599	0.856-0.864	0.987-0.991	0.855-0.863	0.987-0.991	0.855-0.863	0.987-0.991	0.856-0.864	0.983-0.987
AF442613 Russia (RUS), M32750, L08804, Z84204, AB433846, AB433848, AB433845, Z30708, Z30706, Z30707, Z21497	0.846-0.863	0.970-0.978	0.845-0.862	0.970-0.978	0.845-0.862	0.970-0.978	0.846-0.863	0.966-0.974
Z30702 Finland (FIN), Z30704, Z30705, GU808825, GU808824, AJ314597, Z46942, Z69985, JQ319171, JQ319163, JQ319166, JQ319167, JQ319168, JQ319161, JQ319164, JQ319170, JQ319162, JN831950, HE801633	0.815-0.832	0.928-0.966	0.817-0.831	0.928-0.966	0.814-0.831	0.928-0.966	0.815-0.832	0.924-0.962
AJ888751 Austria (ALAD), AJ888752	0.804	0.940	0.805	0.940	0.803	0.940	0.804	0.936
KC676609 Croatia (ALAD), KC676613	0.805-0.807	0.949-0.953	0.804-0.805	0.949-0.953	0.804-0.805	0.949-0.953	0.805-0.807	0.945-0.949
FN377821 Hungary (ALAD), FN377822	0.810-0.807	0.957	0.808-0.811	0.957	0.805-0.808	0.957	0.810-0.807	0.953

AJ314600 Balkan (ALAD),<br>AJ314601	0.810-<br>0.807	0.949	0.808-<br>0.811	0.949	0.805-<br>0.808	0.949	0.810-<br>0.807	0.945
GQ339483 Sweden (S-SCA),<br>Q339484, GQ339485, GQ339487, GQ339486, AJ223376	0.817-<br>0.841	0.974-<br>0.966	0.818-<br>0.842	0.974-<br>0.966	0.817-<br>0.841	0.974-<br>0.966	0.817-<br>0.841	0.970-<br>0.962
AJ223368 Norway (S-SCA),<br>AJ223369	0.817-<br>0.818	0.962	0.817-<br>0.818	0.962	0.818-<br>0.819	0.962	0.817-<br>0.818	0.957
EU439968 Germany (CE),<br>EU439969, EU439972, DQ016430, DQ016432, Y954722,<br>JN696358, KJ994776, JN696373, JN696372, JN696371,<br>JN696374, JN696376	0.805-<br>0.843	0.970-<br>0.957	0.804-<br>0.845	0.970-<br>0.957	0.807-<br>0.842	0.970-<br>0.957	0.805-<br>0.842	0.966-<br>0.953
U22423 Belgium (CE),<br>J277075, AJ277076, AJ277034, AJ277033, AJ277032,<br>AJ277031, AJ277030	0.821-<br>0.797	0.957-<br>0.940	0.822-<br>0.798	0.957-<br>0.940	0.819-<br>0.796	0.957-<br>0.940	0.821-<br>0.797	0.953-<br>0.936
AM695638 France (CE),<br>KT247592, KT247594, KT247595, KT247596, KT247597	0.825-<br>0.807	0.962-<br>0.957	0.824-<br>0.808	0.962-<br>0.957	0.824-<br>0.805	0.962-<br>0.957	0.825-<br>0.805	0.957-<br>0.953
GQ339476 Sweden (N-SCA),<br>GQ339477, AM746297, AM746298, AM746310, AM746311,<br>AM746315, AM746316, AM746317, AM746318, AM746319,<br>GQ339478, GQ339480, GQ339482, GQ339473, GQ339474,<br>GQ339481, GQ339479, AM746320, AM746322, AM746324,<br>AM746325, AM746328, AM746329, AM746330, AJ223371,<br>AJ223374, AJ223375, AJ223380, Z48586, AM746332,<br>AM746331, AM746333, AY526219, U14137, AJ223377	0.828-<br>0.797	0.974-<br>0.949	0.829-<br>0.796	0.974-<br>0.949	0.828-<br>0.798	0.974-<br>0.949	0.828-<br>0.797	0.970-<br>0.945
AJ238791 Denmark (DAN),<br>AJ278092, AJ278093	0.805-<br>0.786	0.953-<br>0.932	0.804-<br>0.787	0.953-<br>0.932	0.804-<br>0.784	0.953-<br>0.932	0.805-<br>0.786	0.949-<br>0.928

PUUV lineage affiliation is given at the end of sequence names: (ALAD) Alpe-Adrian lineage, (CE) Central European lineage, (DAN) Danish lineage, (FIN) Finnish lineage, (LAT) Latvian lineage, (N-SCA) North-Scandinavian lineage, (RUS) Russian lineage, (S-SCA) South-Scandinavian lineage. PUUV S segment sequences from colony bank voles 2, 4, 5, 8, 13, 14, 15, 17 and 18 were identical on nucleotide sequence level and therefore excluded.

(b) Reference sequences of the M segment	PV798424 1gen14 Kraków Poland (RUS)		PV798425 4gen14 Kraków Poland (RUS)	
	nt	aa	nt	aa
PV798424 1gen14 Kraków lab. colony Poland (RUS)	-	-	**0.998**	1
PV798425 4gen14 Kraków lab. colony Poland (RUS)	**0.998**	1	-	-
KX815397 KS14/118 Teleśnica Oszwarowa Poland (RUS)	0.987	0.995	0.985	0.995
KX815398 KS14/121 Teleśnica Oszwarowa Poland (RUS)	**0.998**	1	0.996	1
KF906513 KS13/855 Mikołajki Poland (LAT)	0.804	0.922	0.805	0.922
AF442614 Russia (RUS), M29979, Z84205, AB433850, AB433851, AB433852	0.865-0.843	0.985-0.970	0.867-0.844	0.985-0.970
Z70201 Finland (FIN), JQ319173, JQ319175, JQ319172, JQ319174, JN831951, NC_005223, HE801634	0.857-0.810	0.966-0.946	0.859-0.812	0.966-0.946
KC676630 Croatia (ALAD), KC676632, KC676634	0.826-0.823	0.922-0.917	0.828-0.825	0.922-0.917
DQ518223 Germany (CE), DQ518225, DQ518236, DQ518237, DQ518219, DQ518218, AJ238778, DQ518217, DQ518215, DQ518213, DQ518222, DQ518221, DQ518227, KJ994777	0.822-0.791	0.932-0.917	0.823-0.792	0.932-0.917
U22418 Belgium (CE)	0.802	0.927	0.804	0.927
KT247603 France (CE), KT247602, KT247598, KT247599, KT247600, KT247601	0.813-0.796	0.927-0.893	0.815-0.797	0.927-0.893
Z49214 Sweden (N-SCA), AY526218, U14136	0.818-0.792	0.941-0.902	0.794-0.820	0.941-0.902

PUUV lineage affiliation is given at the end of sequence names: (ALAD) Alpe-Adrian lineage, (CE) Central European lineage, (FIN) Finnish lineage, (LAT) Latvian lineage, (N-SCA) North-Scandinavian lineage, (RUS) Russian lineage. PUUV M segment sequences from colony bank voles 2, 5, 6, 8, 9, 13, 14 and 17 were identical on nucleotide level and therefore excluded.

(c) Reference sequences of the L segment	PV798426<br>1gen14 Kraków<br>Poland (RUS)	
	nt	aa
PV798426 1gen14 Kraków lab. colony Poland (RUS)	-	-
KX815400 KS14/118 Teleśnica Oszwarowa Poland (RUS)	0.995	1
KX815401 KS14/121 Teleśnica Oszwarowa Poland (RUS)	**0.997**	1
KX815399 KS13/855 Mikołajki Poland (LAT)	0.866	0.978
OL355107 Lithuania (LAT)	0.868	1
EF405801 Russia (RUS),<br>OP561837, OP561846, OP561849, OP561852	0.873-0.851	1-0.963
JN831945 Finland (FIN),<br>HE801635, MT024595, MT024592	0.875-0.861	1-0.992
MT514291 Germany (CE);<br>MT514293, MW656500, MN639749	0.868-0.851	0.985-0.956
KT247604 France (CE),<br>KT247605, KT247606, KT247607, KT247608, KT247609	0.863-0.832	0.970-0.941
AY526217 Sweden (N-SCA),<br>MN832779	0.873-0.854	0.978-0.970

PUUV lineage affiliation is given at the end of sequence names: (CE) Central European lineage, (FIN) Finnish lineage, (LAT) Latvian lineage, (N-SCA) North-Scandinavian lineage, (RUS) Russian lineage. On nucleotide level all PUUV L segment sequences from colony bank voles were identical, therefore only the sequence from individual 1 was included.
