# Supplementary material for: Maternal antibody-mediated elimination of a Puumala hantavirus outbreak in a bank vole colony
Source: PLoS Pathog. 2026 May 29;22(5):e1013693. doi: 10.1371/journal.ppat.1013693 (PMC13241010; doi:10.1371/journal.ppat.1013693)
Supplement: S1 Protocols — (PDF) [file ppat.1013693.s005.pdf]

Protocols of the RNA analyses performed during the PUUV eradication program

Prepared by Mateusz Konczal (15.07.2013)

I. Protocol: RNA extraction using RNAzol

Introduction

This protocol is a streamlined version of the protocol available at http://www.mrcgene.com/rnazol.htm tailored for the typical applications in our lab. For full explanation and modifications see the web page

Materials and reagents

- ethanol 96-100%
- ethanol 75%
- RNAse free water must be used for preparation of ethanol solutions
- RNAse free pipette tips and eppendorf tubes, essential! both 1.5 and 2 ml. We buy RNAse free plasticware
- DEPC-treated metal homogenizer tips. They should be soaked (min 12 h) in water containing 0.1% DEPC, and then dry in the drier, individually wrapped in the aluminium foil for ca. 24 h.

Introductory remarks

Disposable gloves MUST be used at all times and changed frequently, human hands are a major source of RNAses

PROCEDURE

1. Use 600µl of RNAzol.
2. a) For samples of liver: take the piece of liver (preserved in RNAlater), place in RNAzol and homogenise using a motor homogenizer with diethyl pyrocarbonate (DEPC)-treated metal tips (2 × 15 s), and store for 15 minutes.
   b) For the samples of saliva or dust: add 240 µl sample solution (buffor with RNA) to RNAzol. Shake the resulting mixture vigorously for 15 seconds and store for 15 minutes.
3. Centrifuge samples at 12,000 g for 15 min. Following centrifugation, DNA, proteins and most polysaccharides form a semisolid pellet at the bottom of the tube. The RNA remains soluble in the supernatant.
4. Transfer 75% of total supernatant volume to a new tube, leaving a layer of the supernatant above the DNA/protein pellet. Precipitate RNA by mixing the transferred supernatant with 75% ethanol (0.4 ml of 75% ethanol (v/v) per 1 ml of supernatant).
5. Store samples for 10 min
6. Centrifuge samples at 12,000 g for 8 min. RNA precipitate forms a white pellet at the bottom of a tube.
7. Remove supernatant by pouring
8. Add 0.5 ml of 75% ethanol

9. Centrifuge at 8,000 g for 3 min
10. Remove the alcohol solution by pouring.
11. Add 0.5 ml of 75% ethanol
12. Centrifuge at 8,000 g for 3 min
13. Remove the alcohol solution by pouring and then using a micropipette.
14. Dissolve the RNA pellet, without drying, in 50-100 ul of water and store on ice
15. Heat to 65°C for 3 min to disrupt secondary structures. Immediately place on ice.
16. Store at -70°C
17. RNA concentration may be measured using NanoDrop or Tecan

II. Protocol: RT-qPCR for PUUV identyfication

Materials and methods

- QuantiTect Probe RT-PCR Kit
- Primers (Primer F: gtgcaccagatcgrtgtcc, Primer R: yarctctgccatccctgca; both 5'->3')
- TaqMan probe (ccaacatgyatttatg)
- RNase-free water

Thaw reagents at the room temperature. Mix well on vortex or by pipetting before usage. It is crucial step for successful procedure.

PROCEDURE

1. Prepare MM for all samples (1.05–1.1 x n, where n is number of samples used). Use per one sample:

Master Mix (2x)	10 µl
Primer F (100ng/µl)	0.08 µl
Primer R (100ng/µl)	0.08 µl
TaqMan	0.04 µl
RT Mix	0.20 µl
H_2O	4.6 µl
**Sum**	**15 µl**

2. Add to each well in the plate 15 µl MM. Remember about negative and positive control.
3. Add to each well 5 µl of RNA sample.
4. Start qPCR machine with the following conditions:

1. 50°C	30 min
2. 95°C	15 min
3. 94°C	15 sec
4. 59°C	60 sec

Repeat steps 3-4: 45x
