# Supplementary material for: Maternal antibody-mediated elimination of a Puumala hantavirus outbreak in a bank vole colony
Source: PLoS Pathog. 2026 May 29;22(5):e1013693. doi: 10.1371/journal.ppat.1013693 (PMC13241010; doi:10.1371/journal.ppat.1013693)
Supplement: S1 Raw images — (PDF) [file ppat.1013693.s006.pdf]

Drewes et al.: S1 Raw images

Raw images of agarose gels from the RT-PCR analyses targeting the segment S of PUUV

A) The whole gel

Samples 1-14

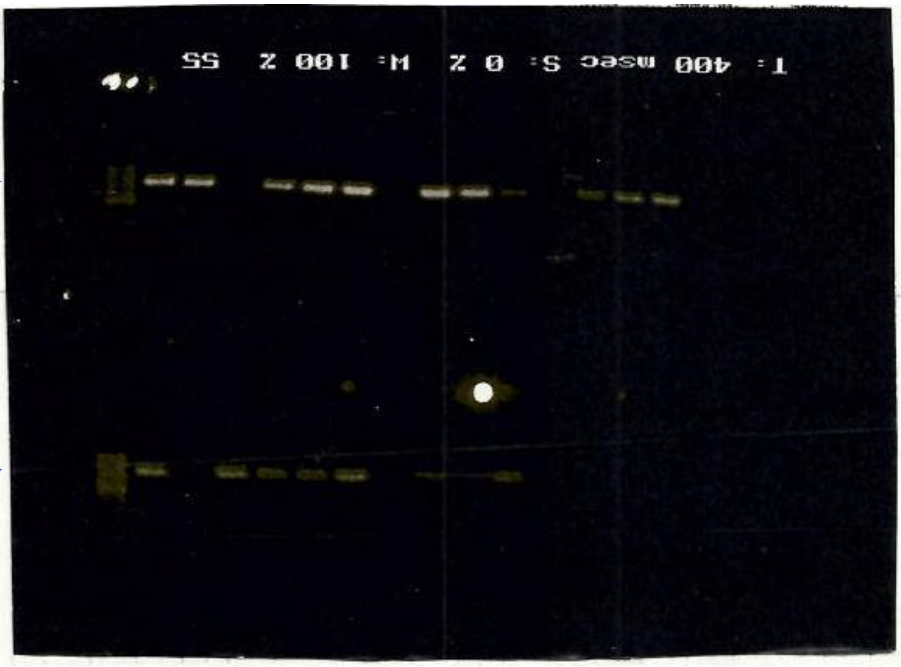

Samples 15-22 and positive and negative control

B) Parts of the same gell phoptographed separately, with sample identity

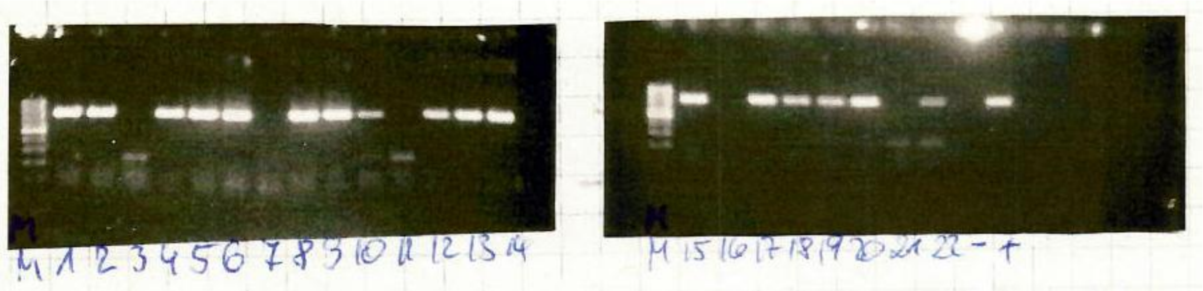

M 1 2 3 4 5 6 7 8 9 10 11 12 13 14   M 15 16 17 18 19 20 21 22 NC PC

M: marker of molecular mass

NC: negative control (DEPC-treateed RNase-free water)

PC: positive control (RNA isolated from cell culture propagated PUUV strain Sotkamo)
